# Sanitization of food-grade materials: Combined effect of an enzymatic product and two chemical sanitizers on *Listeria monocytogenes* biofilms

**DOI:** 10.3934/microbiol.2026018

**Published:** 2026-06-30

**Authors:** Serena Iannone, Francesco Blasi, Nicoletta Scaramuzza, Barbara Franceschini, Massimo Cigarini, Elettra Berni

**Affiliations:** 1 Department of Biotechnology and Biosciences BtBs, University of Milan-Bicocca, Piazza della Scienza 2, 20126 Milan, Italy; 2 Department of Food and Drug, University of Parma, Parco Area delle Scienze 31/A, 43124 Parma, Italy; 3 Microbiological Laboratory, Stazione Sperimentale per l'Industria delle Conserve Alimentari (SSICA)—Research Foundation, Viale F. Tanara 31/A, 43121 Parma, Italy; 4 Food Safety Division, Stazione Sperimentale per l'Industria delle Conserve Alimentari (SSICA)—Research Foundation, Viale F. Tanara 31/A, 43121 Parma, Italy

**Keywords:** biofilm, sanitization, ammonium quaternary salts, QAC, ethanol, enzymes, *Listeria monocytogenes*

## Abstract

In industrial practice, different food-grade materials could be subjected to contamination by microorganisms that are responsible for the spoilage of foods. The chemical sanitizers used to inactivate microbes on the food-grade surfaces have proved effective on vegetative cells, but may not be successful on biofilms, thus encouraging food companies to search alternative ways to eradicate these microbes. For this reason, in this work, two commercially available chemicals based on quaternary ammonium salts (QAC) and alcohols were tested alone or in combination with an enzymatic product against seven-day-old biofilms of three *Listeria monocytogenes* strains on stainless steel (SS) or poly-tetra-fluoro-ethylene (PTFE). When the sanitizers were applied without any pre-treatment, with the QAC, no survivors were detected after 10 minutes at a concentration of 0.75–1.0% on SS; on PTFE, it was necessary to increase the concentration to 1.0% to obtain a total inactivation of the sessile cells for all the tested strains. The use of the alcoholic product enabled us to obtain total inactivation of the sessile cells at a concentration of 50% for contact times greater than 2.5–5 minutes on SS; on the contrary, on PTFE, it was necessary to use the undiluted sanitizer for 10 minutes for all the tested strains. When the enzymatic solution was used as a pre-treatment, the achieved logarithmic reductions were significantly higher than the ones obtained when using the sanitizing products alone: In half of the cases, this combination enabled us to obtain a total inactivation on the biofilm-forming cells.

## 1. Introduction

In food industries, working surfaces and equipment used by operators could be subjected to contamination by microorganisms that are responsible for the spoilage of foods. In this context, mostly yeasts and bacteria have the ability to irreversibly attach to the above-mentioned surfaces that can be made of stainless steel, polystyrene, hydroxyapatite, glass, or rubber [1]–[4]. Such phenomenon can be influenced by electrostatic forces of attraction, steric forces, and hydration forces, and by the structural characteristics of the involved materials, which could give rise to the formation of complex superstructures called biofilms. The transition from a planktonic growth to a biofilm usually happens as a response to environmental changes such as variations in nutrient availability, temperature, pH, oxygen levels, or the presence of sub-inhibitory antibiotics. These stress-inducing conditions trigger genetic and molecular regulatory networks that translate signals to concerted gene expression changes, thus enabling microbes to survive in adverse environments [5]. The formation of a biofilm typically includes five major stages: (a) The adhesion of planktonic cells to some surface via Brownian motions, flagella-driven mobility, and Van der Walls interactions; (b) their permanent anchorage due to the action of flagella or fimbriae; (c) the complete maturation of a structure composed by cells duplicating themselves and producing a highly compartmentalized extracellular matrix limiting the diffusion of external molecules; (d) the partial break-down of the polysaccharidic wall and the subsequent dispersal of dead cells and motile cells; and (e) the restart of the process [6]. The complexity of the above-mentioned superstructure is responsible for the increased resistance of biofilms to mechanical, chemical, and physical stresses [7], and desiccation [8], if compared to planktonic cells. Therefore, the sanitizers usually effective in removing a wide range of microbes from food-grade surfaces (e.g., chlorine, iodine, alcohols, and quaternary ammonium salts, rather than formulas containing hydrogen peroxide or peracetic acid) are not successful on biofilms. This is particularly true for the hard-to-clean area such as drains, floors, conveyor belts, scratches, and joints [9]. Furthermore, the emerging issues of the increased microbial tolerance and the need to avoid residues after each sanitizing process have highlighted the need for alternative strategies to counteract biofilm formation or eradicate them. Alternative methods, including plant-derived essential oils, lactic acid bacteria, and bacteriophages, have begun to be tested against a wide range of microorganisms [10],[11]. Among such innovative strategies, enzymes are gaining an increasing interest, since they are effective on growing and pre-existing biofilms, relatively low concentrations are required to achieve high specificity and efficacy, and they do not seem to be affected by an antibiotic-resistance-like mechanism [12],[13]. On the basis of the chemical reaction they catalyze, enzymes are variably effective against biofilms formed by spoiling bacteria such as *Pseudomonas fluorescens* [14],[15], *Enterococcus faecalis* [16], and *Staphylococcus epidermidis* [17], or by pathogens such as *Escherichia coli* O157:H7, *Pseudomonas aeruginosa*, *Staphylococcus aureus*, *Salmonella enterica* subsp. *enterica* serotype Enteritidis, *Salmonella enterica* subsp. *enterica* serotype Typhimurium, *Bacillus cereus*, and *Listeria monocytogenes* [18]–[20]. *L. monocytogenes* is of particular concern for food industries due to its great adaptability to the environmental conditions registered in food plants [21], to its infectiveness causing listeriosis, which makes it a food safety criterion in Regulation (EC) No. 2073/2005 [22], and to its high-binding capacity to food contact surfaces [23]. The enzymes known to be effective against *L. monocytogenes* biofilms are DNases, protases, α-amylases, pronases, and cellulases, often showing a synergistic effect if used in combination [18],[24],[25]. The food-grade materials where enzymes are partially or totally effective against single- or dual-species biofilms containing *L. monocytogenes* are stainless steel [18],[24]–[28], polypropylene [18], glass [28],[29]. For these reasons, our aim of this study was to assess the effectiveness of two commercially available chemicals based on quaternary ammonium salts (QAC) and alcohols (ALC), alone or in combination, with an enzymatic solution commercially available. The work was targeted against three strains of *L. monocytogenes* embedded in the interconnected and cohesive microenvironment of mature biofilms. Stainless steel (SS) and poly-tetra-fluoro-ethylene (PTFE) were the food-grade materials chosen, since the former is considered the “backbone” of food machineries, and the latter is frequently used for its non-stick properties in components requiring low friction or high heat resistance.

## 2. Materials and methods

### 2.1. Bacterial strains and suspensions

*L. monocytogenes* was chosen not only for its strong association with food surfaces and products, but also for its pathogenicity to humans that poses a significant threat to the food industry as it is ubiquitous, able to grow over a wide range of temperatures (from 0 to 45 °C, with an optimum of 30–37 °C), and multiply on most non-acidic foods [30],[31]. In this work, the following strains were tested:

- *L. monocytogenes* Scott A (CIP 103575; CECT 5672; ATCC 49594), isolated from a human host involved in a listeriosis outbreak.

- *L. monocytogenes* SSICA CO1, isolated from a knife used for working operations in a food industry in Parma (Italy).

- *L. monocytogenes* SSICA AF1, isolated from a slicer in a meat industry in Parma (Italy).

Bacterial suspensions were prepared from strains preserved at −20 °C in porous beads (Cryoinstant; WVR, Milan, Italy). One bead for each strain was separately added to 10 mL of Brain Heart Infusion (BHI; Oxoid, Cambridge, UK) broth and incubated at 30 °C for 24 h. After incubation, 0.1 mL of each bacterial suspension was re-suspended in 10 mL of sterile BHI broth and incubated at 30 °C for 24 h before being used for the biofilm formations.

### 2.2. Enzymatic solution and sanitizers

QAC (10–20% alkyl-dimethyl-benzyl-ammonium chloride; sodium hydroxide) and ALC (50–75% ethanol; 1–5% isopropyl alcohol) were applied to SS or PTFE tiles at different contact times (2.5, 5.0, 7.5, 10.0 min). QAC was used at concentrations from 0.25 to 1.0% (v/v); ALC was used at concentrations from 50% to 100% (v/v), depending on the tested material.

The enzymatic product used was commercially available under the trademark MIDA ENZY 1002 (Christeyns Italia, Milan, Italy). It was applied on SS or PTFE tiles at a concentration of 3.0% for 30 min, according to the manufacturer's operating instructions, before tiles were treated with both sanitizers at time/concentration that did not prove effective in tests with sanitizers only.

### 2.3. Biofilm formation and detachment

Each bacterial suspension was centrifuged at 5,000 rpm for 10 min, and the pellet was resuspended in 10 mL of a quarter-strength Ringer solution (2.25 g/L sodium chloride; 0.105 g/L potassium chloride; 0.12 g/L calcium chloride 6H_2_O; 0.05 g/L sodium bicarbonate) to obtain a final bacterial concentration of about 8 Log CFU/mL. SS tiles and PTFE tiles (15 × 20 × 1 mm) were used as germ carriers after being sterilized at 121 °C for 15 min. The biofilm formation and detachment protocols were optimized by carrying out preliminary tests where two incubation temperatures (15 or 30 °C) and three removal techniques (a. Vortexing for 2 min; b. sonication for 3 min; and c. vortexing for 2 min + sonication for 3 min + vortexing for 2 min) were tested (Table 1).

**Table 1.** Mean counts (Log CFU/tile ± standard deviation) after removal of biofilms formed by *L. monocytogenes* (Lm) at 15 °C or 30 °C on SS or PTFE tiles.

*SS*
Detachment procedure	Incubation temperature (°C)	Lm Scott A	Lm SSICA AF1	Lm SSICA CO1
a.	15	4.90 ± 0.32	6.01 ± 0.37	6.12 ± 0.19
	30	5.00 ± 0.42	6.51 ± 0.18	6.91 ± 0.29
b.	15	5.42 ± 0.42	6.28 ± 0.23	5.99 ± 0.27
	30	6.25 ± 0.48	7.07 ± 0.19	6.47 ± 0.61
c.	15	6.50 ± 0.42	6.66 ± 0.16	6.32 ± 0.25
	30	7.07 ± 0.04	7.66 ± 0.34	6.69 ± 0.01
*PTFE*
Detachment procedure	Incubation temperature (°C)	Lm Scott A	Lm SSICA AF1	Lm SSICA CO1
a.	15	6.30 ± 0.05	6.61 ± 0.21	6.25 ± 0.30
	30	6.81 ± 0.11	7.05 ± 0.24	7.41 ± 0.29
b.	15	6.47 ± 0.15	6.52 ± 0.32	7.02 ± 0.12
	30	7.23 ± 0.13	7.53 ± 0.13	8.09 ± 0.22
c.	15	6.96 ± 0.07	7.03 ± 0.46	7.25 ± 0.07
	30	8.03 ± 0.02	8.09 ± 0.32	8.29 ± 0.38

Detachment procedures: a. vortexing (2 min); b. sonication (3 min); c. vortexing (2 min) + sonication (3 min) + vortexing (2 min).

*Adhesion phase*. Each tile was separately immersed in a screw-cap test tube containing 10 mL of the quarter-strength Ringer solution, which was previously inoculated. It was then incubated at 30 °C up to 3 h, with the attempt to provide good adhesion to the selected material, as done by Blasi et al. [6], transferred in a screw-cap test tube containing 10 mL of sterile BHI, and incubated at 30 °C for 3 days.

*Drying phase*. After the adhesion phase, each tile was collected from the inoculated BHI and placed on a Petri dish under a laminar flow cabinet to dry the biofilm. After the complete desiccation of the cellular layer, each tile was re-immersed in 10 mL of sterile BHI and incubated again at 30 °C for 3 days.

*Treatment phase*. After a 6-day incubation, each tile was collected from the culture broth and washed with 10 mL of a peptone salt solution (8.5 g/L NaCl, 1 g/L tryptone) added with 0.1% (w/v) Polysorbitan Tween 80 (Biolife, Milan, Italy).

When only the sanitizers were used, each washed tile was immersed in the sanitizer for the selected contact time and then directly moved into a sterile screw-cap tube containing 10 mL of a neutralizing solution (1.0 g/L peptone; 8.5 g/L sodium chloride; 3.0 g/L soy lecithin; 5.0 g/L sodium thiosulfate).

**Figure 1.**
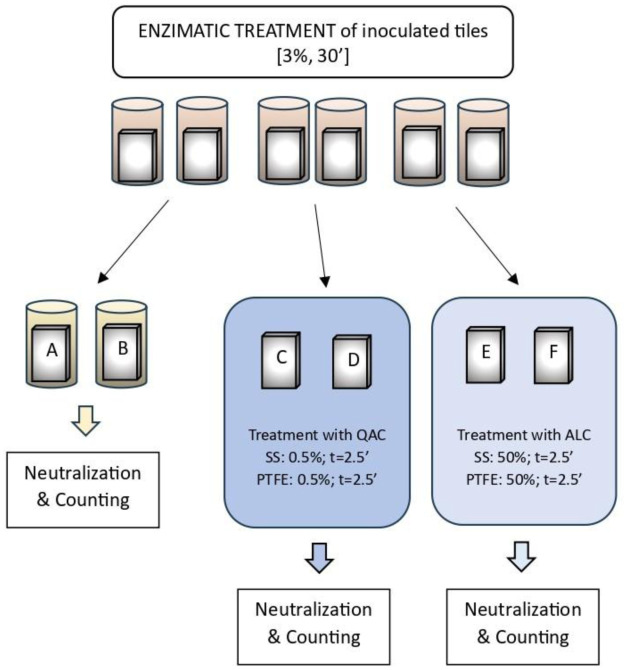
Graphical elaboration of the operative steps carried out to test the effectiveness of MIDA ENZY 1002 and sanitizers on SS or on PTFE tiles covered with *Listeria monocytogenes* biofilms. After the enzymatic treatment, tiles A and B were directly transferred into the neutralizing solution and counted; tiles C and D were transferred into the QAC solution (0.5%, 2.5 minutes) before being neutralized and counted; and tiles E and F were transferred into the ALC solution (50%, 2.5 minutes) before being neutralized and counted.

When the enzymatic product was preliminarily used, each washed tile was immersed in the enzymatic solution (3%, 30 minutes) and then processed in 3 ways (Figure 1):

- One third of the tiles was directly transferred to the neutralizing solution without any rinsing.

- One third of the tiles was directly transferred to the QAC solution (0.5%, 2.5 minutes) before being transferred into the neutralizing solution without any rinsing.

- One third of the tiles was directly transferred to the ALC solution (50%, 2.5 minutes) before being transferred into the neutralizing solution without any rinsing.

In all cases, the neutralizing solution was left to react for at least 15 min before the detachment and counting phases. The absence of toxic effects by the neutralizing solution and the validation of our dilution/neutralization method were positively checked, according to the UNI EN 13697 [32].

*Detachment phase*. The detachment of the biofilm was carried out by vortexing each test tube with an IR apparatus (Starlab, Milan, Italy) for 2 min, sonicating it with a SONOMATIC® ultrasound apparatus (Langford Ultrasonics, Manchester, UK) at a frequency of 40 kHz for a period of 3 min, and vortexing it again for 2 min. Sterile glass microbeads were added to the neutralizing solution to optimize the biofilm removal.

*Counting phase*. Decimal dilution or filtration counts were carried out on Tryptone Soy Agar (TSA; OXOID, Cambridge, UK) and incubated at 30 °C for 48 h.

For each test, two inoculated tiles, marked as “positive controls”, were directly immersed in the neutralizing solution and counted to enable a comparison with the treated tiles.

Each time/concentration combination was tested in duplicate and repeated at least two times.

### 2.4. Statistical analysis

Microsoft® Excel® 2021 MSO (Microsoft, Redmond, WA, USA) was used to elaborate microbial counts that were converted into logarithmic values and to detect statistical differences in the formation/detachment protocol by means of a One-way Analysis of Variance (ANOVA) whose significance was expressed at the 99% confidence level (α = 0.01) (Table S1).

Logarithmic Count Reductions (LCR) were calculated according to the formula reported below (Eq 1):

$$\text{LCR}={\text{Log}}_{10}\text{ number of initial cells }\left(\text{N}0\right)-{\text{Log}}_{10}\text{ number of surviving cells }\left(\text{Nf}\right)$$ (1)

OriginPro 2024 (OriginLab Corporation, Northampton, MA, USA), a data analysis and graphing software for drawing response surfaces, was used to contour the inactivation conditions on SS and PTFE inoculated tiles and to calculate statistical differences between techniques, strains, or materials by means of a Two-way Analysis of Variance (ANOVA), whose significance was expressed at the 99% confidence level (α = 0.01) (Table S2).

## 3. Results and discussion

### 3.1. Biofilm formation and detachment

The structure of the biofilms grown on SS and PTFE is shown for the tested strains in Figure S1.

In the preliminary tests (Table 1), the search for the best formation and detachment protocol enabled us to register highly variable concentrations of microbes (4.90–8.29 Log CFU/tile), but very low standard deviations (0.01–0.61). The former could be attributed to the intra-specific variability occurring among microbes; the latter to the stable and reproducible system of the biofilm.

As stated by other researchers, the use of a quarter-strength Ringer solution seemed effective in reinforcing the formation of *L. monocytogenes* biofilm [33]. This was probably due to the role played by Ca^++^ ions in the adhesion of cells to surfaces, as it is involved in non-specific interactions such as the neutralization of the electrical double-layer between cell and substratum surface and the adhesive interactions that cannot be replaced by other cations [34].

The elective temperature for the optimal growth of the tested strains was 30 °C, and the combination “vortexing + sonication + vortexing” was the best method to detach and disaggregate the bacterial biofilms formed.

When comparing the temperatures, the bacterial counts at 15 °C were significantly lower than those obtained from tiles incubated at 30 °C (α = 0.01). These results agree with the literature, where *L. monocytogenes* was able to adhere and form biofilms on different surfaces regardless of temperature; below 30 °C, the biofilm levels were significantly higher than those obtained at 37 °C [35],[36]. When comparing the detachment techniques, no significative differences were registered when comparing (a.) and (b.), or (b.) and (c.), whereas there was a significative difference when comparing (a.) and (c.) (Table S1). The use of combined techniques to detach cells agreed with the results obtained by Mandakhalikar et al. [37], who observed that a “vortexing-sonication-vortexing” method extracts 2.6 to 2.9 times more bacteria from biofilms than other techniques, which are generally used alone [38] or in partial combinations [39].

### 3.2. Treatments with the sanitizers

**Figure 2.**
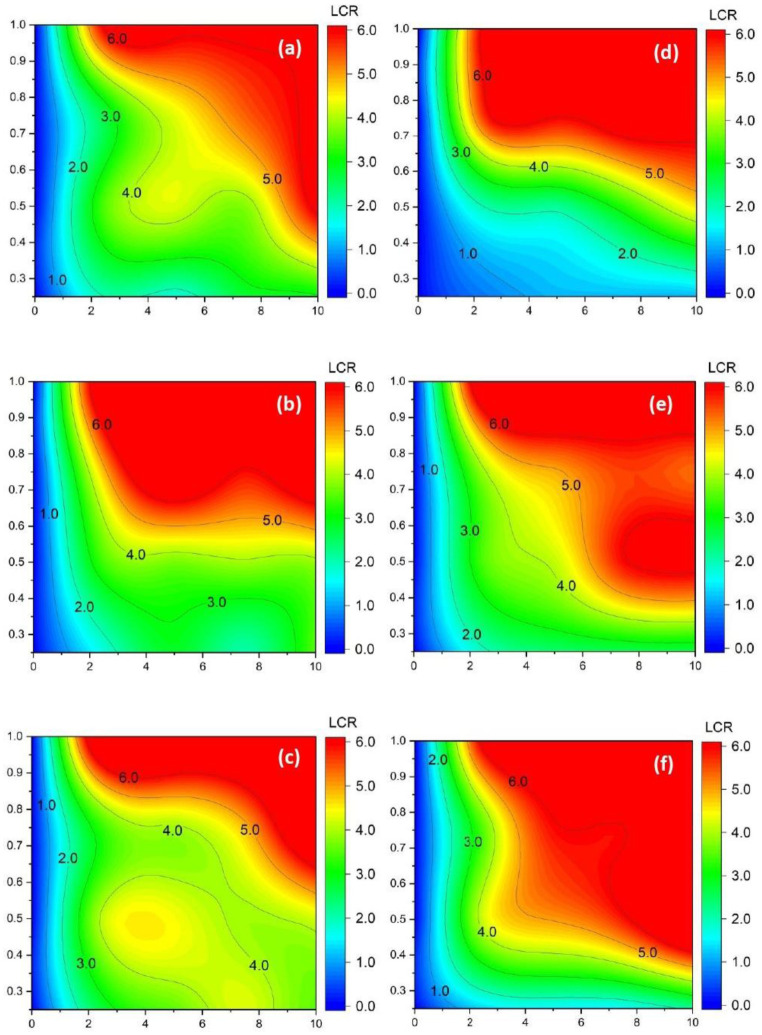
Contour plots showing the LCR obtained by applying QAC to biofilms formed on SS [(a) *L. monocytogenes Scott A*; (b) *L. monocytogenes SSICA CO1*; and (c) *L. monocytogenes SSICA AF1*] or on PTFE [(d) *L. monocytogenes Scott A*; (e) *L. monocytogenes SSICA CO1*; and (f) *L. monocytogenes SSICA AF1*]. On the X Axis, the treatment time (minutes) is reported; on the Y Axis, the concentration of the sanitizer (%, v/v) is reported. On the right side of each graph, a color scale indicating the number of obtained LCR is reported.

**Figure 3.**
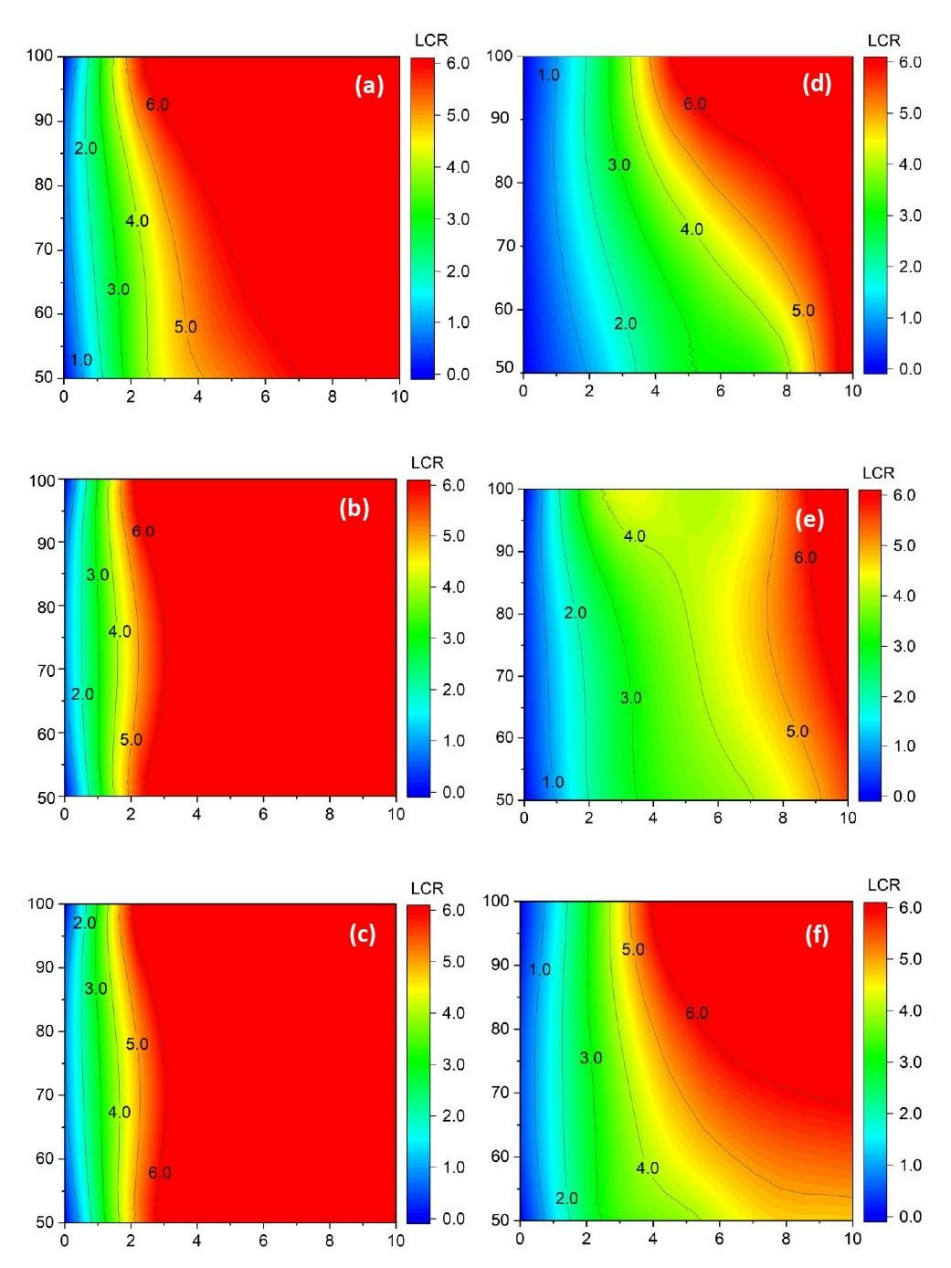
Contour plots showing the LCR obtained by applying ALC to biofilms formed on PTFE [(a) *L. monocytogenes Scott A*; (b) *L. monocytogenes SSICA CO1*; and (c) *L. monocytogenes SSICA AF1*] or on PTFE [(d) *L. monocytogenes Scott A*; (e) *L. monocytogenes SSICA CO1*; and (f) *L. monocytogenes SSICA AF1*]. On the X Axis, the treatment time (minutes) is reported; on the Y Axis, the concentration of the sanitizer (%, v/v) is reported. On the right side of each graph, a color scale indicating the number of obtained LCR is reported.

The results obtained by applying QAC and ALC to inoculated tiles are shown in Figures 2 and 3, respectively. In the contour plots enabling the analysis of relationships between three variables (sanitizer concentration, treatment time, and LCR), a provisional number or logarithmic reductions were given in the interval. Regarding this, since the stronger treatments enabled us to achieve a total inactivation between 6 to 8, depending on the strains and the material tested, the maximum value of the LCR scale was fixed at 6 in both figures to graphically standardize the obtained reductions.

With the QAC-based product, a total cell inactivation after 10 minutes at a concentration of 0.75% (*L. monocytogenes* SSICA AF1, *L. monocytogenes* SSICA CO1), or at a concentration of 1% (*L. monocytogenes* Scott A) was obtained for SS (Figure 2a–c); for PTFE, it was necessary to increase the concentration to 1.0% for all the tested strains due to the survival of some sporadic cells when *L. monocytogenes* SSICA CO1 was considered (Figure 2d–f). The use of the alcoholic product made it possible to obtain a total inactivation at a concentration of 50% for contact times greater than 2.5 minutes (autochthonous strains) or 5 minutes (*L. monocytogenes* Scott A) for SS (Figure 3a–c); on the contrary, for PTFE, it was necessary to use the undiluted sanitizer at the maximum treatment time for all tested strains (Figure 3d–f). The statistical analysis carried out by means of a two-way ANOVA, considering the strains (*L.monocytogenes* Scott A; *L. monocytogenes* SSICA CO1; *L. monocytogenes* SSICA AF1) and the materials (SS; PTFE) as multiple factors enabled us to find a significant difference only for treatments with ALC on different materials (Table S2).

The bacterial biofilms formed on PTFE were more resistant than those formed on SS, which seemed particularly true when the alcoholic product was used. This stronger resistance on plastic materials could be driven by the material hydrophobicity, as well as by its porosity and roughness, thus trapping bacteria within micro-ripples and making it harder for sanitizers to penetrate the crevices. In most cases, an observed tailing in the concentration of the survivors was registered at longer contact times for both sanitizers. Such resistance could be due to the structural conformation of the biofilm (where the exo-polysaccharidic matrix randomly acts as a hurdle to the sanitizer), rather than to the development of a phenotypic resistance (the presence of a sub-population of highly-stressed tolerant cells that proved to survive when subjected to extrinsic factors), as observed for other microorganisms [6],[7]. This phenomenon must be considered in all cases, as it could lead to severe consequences in food plants. As a matter of fact, the survival of even a small fraction of cells enables:

- Accelerated biofilm regrowth (surviving cells use the dead biomass from killed neighbors as a nutrient reservoir, leading to thicker and more metabolically active biofilms than the original).

- Enhanced antimicrobial resistance (tailing often is due to highly resistant sub-populations that can make subsequent disinfection cycles less effective).

- Microbially-induced corrosion (changes in electrochemical properties can give rise to localized pitting and structural failure).

- Operational inefficiency (surviving biofilms in heat exchangers reduce conductive heat transfer and increase friction, leading to higher energy consumption and potential production shutdowns).

### 3.3. Treatments with the enzymatic product and the sanitizers

When used after the application of the enzymatic solution (3%, 30 minutes), the sanitizers were tested at the concentrations that did not prove effective in previous tests, being 0.5% (QAC), or 50% (ALC) for 2.5 minutes (see section 3.2.), to simulate a worst-case scenario. The results obtained are shown in Table 2.

**Table 2.** Mean microbial counts (Log CFU/tile) and LCR obtained on *L. monocytogenes* (Lm) by combining MIDA ENZY 1002 with sanitizers (QAC or ALC).

		SS			PTFE
	Experimental Step	Mean counts	Partial LCR	Total LCR	Mean value	Partial LCR	Total LCR
Lm SSICA CO1	Positive controls	7.61			8.58		
	Cells released in the enzymatic solution	5.90			4.89		
	Tile (enzyme alone)	5.00			6.36		
	Tile (enzyme + QAC)	0.30	4.70	7.31	0.00	6.36	8.58
	Tile (enzyme + ALC)	0.00	5.00	7.61	2.70	3.66	5.88
Lm SSICA AF1	Positive controls	7.35			8.63		
	Cells released in the enzymatic solution	5.86			6.08		
	Tile (enzyme alone)	5.49			6.56		
	Tile (enzyme + QAC)	1.66	3.84	5.69	0.00	6.56	8.63
	Tile (enzyme + ALC)	0.00	5.49	7.35	0.00	6.56	8.63
Lm SCOTT A	Positive controls	7.26			8.59		
	Cells released in the enzymatic solution	5.19			6.28		
	Tile (enzyme alone)	4.81			6.38		
	Tile (enzyme + QAC)	0.69	4.12	6.57	1.64	4.74	6.95
	Tile (enzyme + ALC)	0.00	4.81	7.26	1.76	4.62	6.83

Note. When no survivors were detected by means of decimal counts, a filtration of the total amount (10 mL) of neutralizing solution was further carried out to assess the total inactivation of the detached cells. In this case, the mean value is reported as 0.00 Log CFU/tile. Note. “Partial LCR” indicates the number of reductions obtained if final values were compared to the one registered for the tile after the enzymatic treatment; and “Total LCR” indicates the number of reductions obtained if final values were compared to the one registered for the untreated tile (“positive control”).

As observed by Blasi et al. [6] for *Pseudomonas fluorescens*, the number of cells detected in the enzymatic solution and on the tiles treated with the enzyme never reached the concentration registered for positive controls. This could be a result of the formation of macro-aggregates that we did not succeed to completely disperse into the neutralizing solution, but it could also be possible that the treatment induced *L. monocytogenes* into a Viable-But-Not-Culturable (VBNC) state, which would be reflected in the plate counts. The enzymatic product proved variable effective on biofilm-forming strains. For SS, the combination “enzyme + ALC” enabled us to detect no survivors, whereas some cells were sporadically detected after the “enzyme + QAC” treatments, especially when the autochthonous strain *L. monocytogenes* SSICA AF1 was considered (1.7 Log CFU/tile). For PTFE, total inactivation of the detached cells was obtained with the enzymatic treatment combined with both sanitizers only for the autochthonous strain *L. monocytogenes* SSICA AF1. For *L. monocytogenes* SSICA CO1 and *L. monocytogenes* Scott A, the LCR were in the range 7 to 9 for treatments with the QAC and from 6 to 7 for treatments with the alcoholic product.

**Figure 4.**
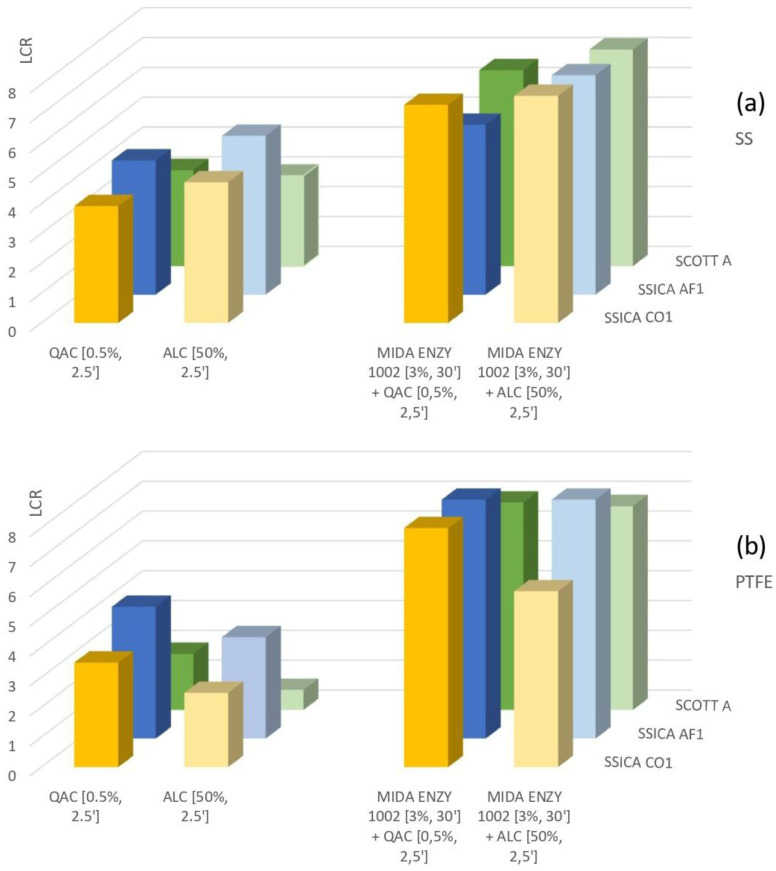
LCR obtained for *Listeria monocytogenes* biofilms treated with sanitizers (left) or with a combination of MIDA ENZY 1002 and sanitizers (right) on SS (a) and on PTFE (b). On the Y axis, a scale indicating the number of reached LCR is reported. On the X Axis, the applied concentration/time combinations are specified for each group of bars. For each strain, different shades of the same color were used, depending on the applied sanitizer.

In general, the achieved LCR were sensibly higher when samples were pre-treated with the enzymatic solution than the ones obtained using the sanitizing products alone (Figure 4a,b) at equal experimental conditions (concentration, treatment time, temperature). In half of the cases, the effect of the enzymatic solution combined with of QAC or ALC enabled us to obtain the highest LCR (that means total inactivation) on the biofilm-forming cells.

### 3.4. Comparisons with literature data

No sanitizing performance criteria have been established for biofilm removal by international regulatory agencies such as the International Organization for Standardization (ISO) or the Association of Official Analytical Collaboration (AOAC) International. Even though 4- to 5-log reductions of planktonic cells are required by either standardized methods or international guidelines when the effectiveness of a sanitizer must be tested, in the case of a biofilm, this target is not sufficient since the incomplete removal of the exo-polysaccharidic matrix and, consequently, the persistence of some embedded cells could lead to a restart of the biofilm formation cycle. Thus, a complete eradication rather than a logarithmic reduction should be pursued. On this aspect, the literature on *L. monocytogenes* biofilm inactivation has flourished in the last decades.

Regarding the sanitizers used in this study, alcoholic products had variable effectiveness: A slight initial reduction (1.0 log CFU/cm^2^) with no further increase in reduction throughout the exposure time was registered when 70% ethanol was used for 30 min on SS and polypropylene (PP), whereas a reduction of 2.5 log CFU/cm^2^ was observed when a combination of a quaternary ammonia compound (0.015%) and isopropyl alcohol (25%) was used on the same materials and for the same treatment times [40]. Moreover, a reduction of about 3 log cycles after 15 min was reported when 75% ethanol was applied to PVC [41]. QAC had varying effects depending on the applied formation protocol, the presence of organic matter, the time/concentration combination, etc. Concentrations lower than 0.1% generally proved only partially effective in *L. monocytogenes* biofilm inactivation, enabling 2.6 log reductions after 10 minutes on SS [42]; 3.6 log reductions on polystyrene after 1 minute [43]; from 2- to 3-log reduction on SS just after a 30-s treatment [44]; or from 3- to 4-log reduction on different materials after a 5-minutes treatment [45]. The same concentrations effectively reduced *L. monocytogenes* by 6 logarithmic cycles for longer treatment times (24 h) in the study by Iñiguez-Moreno et al. [46], or inactivated all biofilm-forming cells after 3 min in the work by Chaves et al. [47]. Such results differed from those obtained in this study, where concentrations up to 0.75% were needed to obtain comparable log reductions, and only 1.0% QAC enabled total inactivation of the treated cells, which then detached from both analyzed surfaces. Such differences could be attributed to the different formation protocols applied and that our biofilms were significantly older and presumably more resistant than those obtained in the cited literature.

Regarding the use of enzymatic products prior to or together with sanitizing treatment, few researchers faced this issue regarding *L. monocytogenes*. The first report was that by Rodriguez-Lopez et al. [27], who investigated the effect of a pronase, a cellulase, and a DNAseI combined with benzalkonium chloride against dual biofilms of *L. monocytogenes* and *E. coli* on SS, highlighting a maximum effect (about 2 LCR) after the application of 400 µg/mL of DNaseI. Analogously, a mixture of tertiary amines, enzymes, and QAC was only partially effective in reducing the microbial loads of dual-species biofilms formed by *L. monocytogenes/Pseudomonas* or *L. monocytogenes/Salmonella* on glass fiber filters: At a concentration equal to 0.085% for 10 min, only 2 LCR were obtained [29]. After approximately the same period of time, a mixture of proteolytic and amylolytic enzymes were used for biofilm removal, alone, or combined with peracetic acid as a disinfectant: For SS, all biofilms were removed after treatments, except the enzymatic treatment alone, which gave only 2 LCR, whereas for PP, the LCR ranged between 0.59 and 5.21 [18]. Similar results were obtained by Mazaheri et al. [24],[26]. Apart from the most commonly investigated enzymes, a lipase mixture from the bacterium *Oceanobacillus* was tested by Nahar et al. [48], who recorded less than 1 LCR in the disruption of a *Listeria* biofilm formed on a glass surface after 1 h of treatment with 150 µL/mL of the above-mentioned mixture. As for the literature data concerning only sanitizing treatments, the above-collected data tend to differ from the ones found in this paper. Apart from the strain-dependent behavior of the *L. monocytogenes* strains tested, the different mechanism of action of the enzymatic formulas could be responsible for such differences, resulting in an overperformance of our enzymatic product.

## 4. Conclusions

The persistence and adaptation abilities of most biofilm-forming microbes complexify their management at an industrial level. Although chemicals have been a widespread strategy to counteract biofilms, new approaches aimed toward sustainability and renewability have begun to play an increasingly important role in the sanitizing processes within food industries. Such new strategies have appeared to overcome the criticisms related to the potential microbial tolerance toward chemical biocides, the possible adverse effects that chemical residues can have on human health, or the reduced power of a chemical product when residual organic matter is present on the materials to be treated. In this perspective, enzymes can be seen as a solution, since they facilitate cell inactivation due to their matrix-disrupting ability without leaving residues. Unfortunately, based on their way of action, they are genus- or species-specific, and this criticism is worsened by the strain-dependent behavior that a microorganism can have when subjected to stress. In addition to this, the contact times that were effective in disrupting the exo-polysaccharidic matrix of the biofilm are, in most cases, not applicable in industrial practice due to tight schedules in the production processes. Finally, since the success of a sanitizing process varies greatly based on the surfaces treated, the use of an enzymatic solution prior to or together with any sanitizing agent should be planned considering the materials in the production environments.

Thus, for the reasons above, as for any traditional sanitizing process, food companies facing biofilm formation problems should practice the combined use of enzymes and sanitizers according to the circumstances. Moreover, it is crucial to understand that surfaces can be simultaneously colonized by more than one microorganism, which strengthens the biofilm, and that a re-growth is always possible over time.

## Use of AI tools declaration

The authors declare they have not used Artificial Intelligence (AI) tools in the creation of this article.


