# Supplementary material for: Sanitization of food-grade materials: Combined effect of an enzymatic product and two chemical sanitizers on Listeria monocytogenes biofilms
Source: AIMS Microbiol. 2026 Jun 30;12(2):422–38. doi: 10.3934/microbiol.2026018 (PMC13370277; doi:10.3934/microbiol.2026018)
Supplement: Supplementary file 1 [file microbiol-12-02-018-s001.pdf]

Microbiology

AIMS Microbiology, 12(2): 422–438.
DOI: 10.3934/microbiol.2026018
Received: 04 November 2025
Revised: 04 June 2026
Accepted: 22 June 2026
Published: 30 June 2026

http://www.aimspress.com/journal/microbiology

Research article

Sanitization of food-grade materials: Combined effect of an enzymatic product and two chemical sanitizers on *Listeria monocytogenes* biofilms

Serena Iannone[1], Francesco Blasi[2], Nicoletta Scaramuzza[3], Barbara Franceschini[4], Massimo Cigarini[4] and Elettra Berni[4,*]

[1] Department of Biotechnology and Biosciences BtBs, University of Milan-Bicocca, Piazza della Scienza 2, 20126 Milan, Italy
[2] Department of Food and Drug, University of Parma, Parco Area delle Scienze 31/A, 43124 Parma, Italy
[3] Microbiological Laboratory, Stazione Sperimentale per l'Industria delle Conserve Alimentari (SSICA)—Research Foundation, Viale F. Tanara 31/A, 43121 Parma, Italy
[4] Food Safety Division, Stazione Sperimentale per l'Industria delle Conserve Alimentari (SSICA)—Research Foundation, Viale F. Tanara 31/A, 43121 Parma, Italy

* **Correspondence:** Email: elettra.berni@ssica.it; Tel: +390521795269; Fax: +390521795218.

Supplementary

Table S1. One-way Analysis of Variance (ANOVA) with a significance at a 99% confidence level (α = 0.01) on analytical techniques tested to optimize the formation/detachment protocol.

ONE-WAY ANOVA (a/b)						
SUMMARY						
Groups	N	Sum	Mean	Variance		
v	24	151,86	6,3275	0,59715		
s	24	160,72	6,696667	0,528423		
ANOVA						
Origin of the variation	SQ	dof	MQ	F	Significance value	F crit
Between groups	1,635408	1	1,635408	2,905912	0,095001553	7,220042
Within groups	25,88818	46	0,562787			
Total	27,52359	47				
ONE-WAY ANOVA (b/c)						
SUMMARY						
Groups	N	Sum	Mean	Variance		
b	24	160,72	6,696667	0,528423		
c	24	173,08	7,211667	0,445093		
ANOVA						
Origin of the variation	SQ	dof	MQ	F	Significance value	F crit
Between groups	3,1827	1	3,1827	6,538568	0,01391946	7,220042
Within groups	22,39087	46	0,486758			
Total	25,57357	47				
ONE-WAY ANOVA (a/c)						
SUMMARY						
Groups	N	Sum	Mean	Variance		
a	24	151,86	6,3275	0,59715		
c	24	173,08	7,211667	0,445093		
ANOVA						
Origin of the variation	SQ	dof	MQ	F	Significance value	F crit
Between groups	9,381008	1	9,381008	18,00158	0,000105672	7,220042
Within groups	23,97158	46	0,521121			
Total	33,35259	47				

Table S2. Two-way Analysis of Variance (ANOVA) with a significance at a 99% confidence level (α = 0.01) on SS and PTFE inoculated tiles, to assess any statistical difference between techniques, strains, or materials.

ANOVA—Two Way Descriptive Statistics material for ALC								
Material								
		N	Mean	SD	SEM	Variance	Missing	NonMissing
SS		6	5.88167	1.8352	0.749219	3.36798	0	6
PTFE		6	4.64667	2.97464	1.21439	8.84851	0	6
Treatment								
		N	Mean	SD	SEM	Variance	Missing	NonMissing
ALC		6	3.26833	1.65898	0.677276	2.75222	0	6
ENZ + ALC		6	7.26	0.905185	0.36954	0.81936	0	6
Overall								
		N	Mean	SD	SEM	Variance	Missing	NonMissing
		12	5.26417	2.44314	0.705273	5.96892	0	12
Interaction								
		N	Mean	SD	SEM	Variance	Missing	NonMissing
SS	ALC	3	4.35667	1.18745	0.685574	1.41003	0	3
	ENZ + ALC	3	7.40667	0.181751	0.104934	0.0330333	0	3
PTFE	ALC	3	2.18	1.38459	0.799396	1.9171	0	3
	ENZ + ALC	3	7.11333	1.39672	0.806398	1.95083	0	3
Overall ANOVA								
		DF	Sum of Squares	Mean Square	F Value	P Value		
material		1	4.57567	4.57567	3.44619	0.100486		
treatment		1	47.8002	47.8002	36.0009	3.23366E-4		
Interaction		1	2.66021	2.66021	2.00355	0.194661		
Model		3	55.0361	18.3454	13.8169	0.00157263		
Error		8	10.622	1.32775				
Corrected Total		11	65.6581					
At the 0.01 level, the population means of material are not significantly different.								
At the 0.01 level, the population means of treatment are significantly different.								
At the 0.01 level, the interaction between material and treatment is not significant.								

Continued on next page

ANOVA—Two Way Descriptive Statistics material for QAC

Material

		N	Mean	SD	SEM	Variance	Missing	NonMissing
SS		6	5.20167	1.58858	0.648534	2.52358	0	6
PTFE		6	6.965	0.714416	0.291659	0.51039	0	6

Treatment

		N	Mean	SD	SEM	Variance	Missing	NonMissing
QAC		6	5.20167	1.58858	0.648534	2.52358	0	6
ENZ + QAC		6	6.965	0.714416	0.291659	0.51039	0	6

Overall

		N	Mean	SD	SEM	Variance	Missing	NonMissing
		12	6.08333	1.49234	0.430801	2.22708	0	12

Interaction

		N	Mean	SD	SEM	Variance	Missing	NonMissing
SS	QAC	3	3.88	0.640937	0.370045	1.41003	0	3
	ENZ + QAC	3	6.52333	0.811008	0.468235	0.0330333	0	3
PTFE	QAC	3	6.52333	0.811008	0.468235	1.9171	0	3
	ENZ + QAC	3	7.40667	0.181751	0.104934	1.95083	0	3

Overall ANOVA

	DF	Sum of Squares	Mean Square	F Value	P Value
material	1	9.32803	9.32803	21.2085	0.0017434
treatment	1	9.32803	9.32803	21.2085	0.0017434
Interaction	1	2.3232	2.3232	5.2821	0.0506064
Model	3	20.9793	6.99309	15.8997	9.85201E-4
Error	8	3.5186	0.439825		
Corrected Total	11	24.4979			

At the 0.01 level, the population means of material are significantly different.

At the 0.01 level, the population means of treatment are significantly different.

At the 0.01 level, the interaction between material and treatment is not significant.

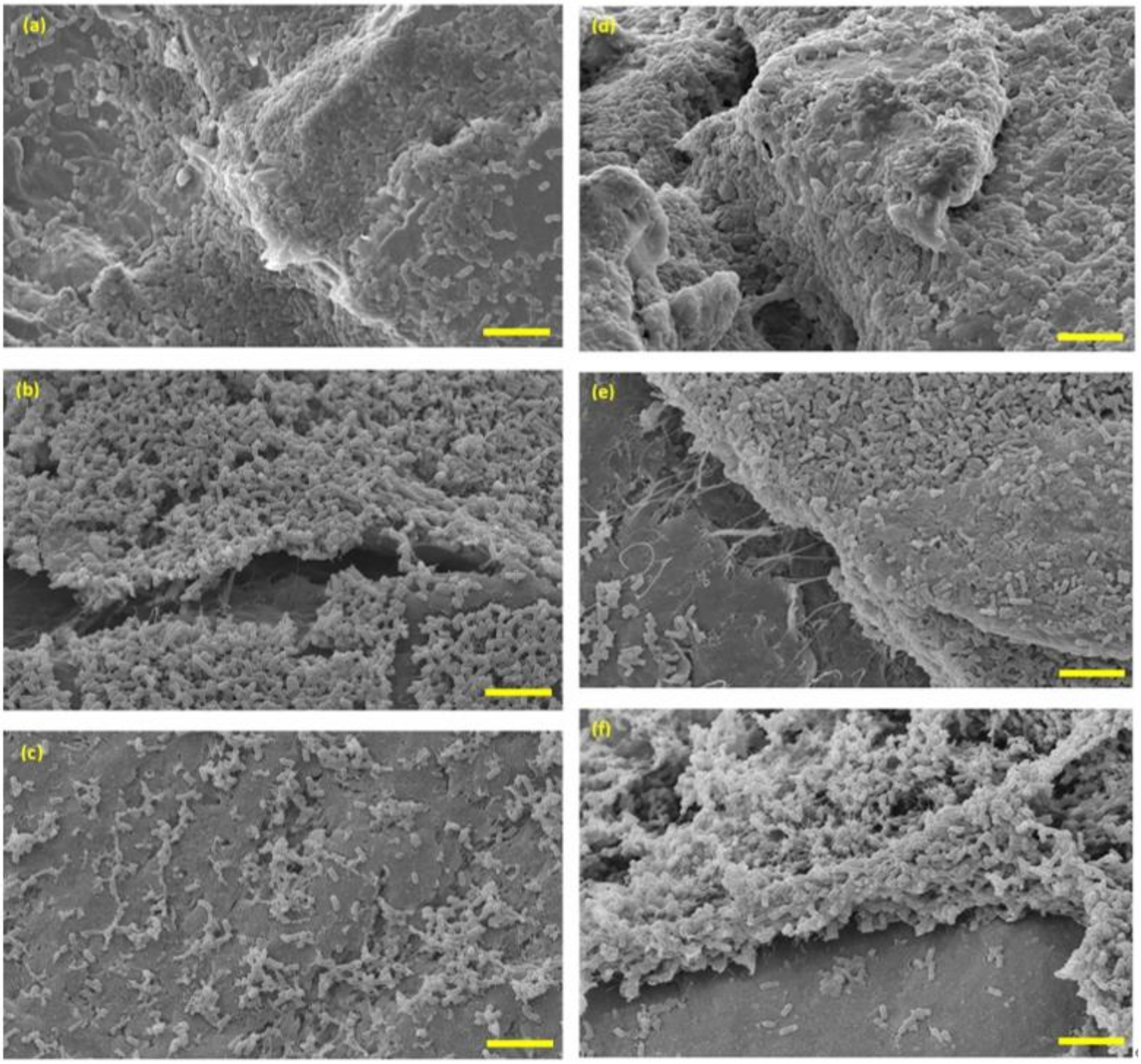

Figure S1. Supplementary material No.3. *Listeria* biofilms on SS [(a) *L. monocytogenes Scott A*; (b) *L. monocytogenes SSICA CO1*; (c) *L. monocytogenes SSICA AF1*] or on PTFE [(d) *L. monocytogenes Scott A*; (e) *L. monocytogenes SSICA CO1*; (f) *L. monocytogenes SSICA AF1*] captured by a Scanning Electron microscope (SEM) at 3,000 magnifications. Scale bar = 5 µm.

AIMS Press

© 2026 the Author(s), licensee AIMS Press. This is an open access article distributed under the terms of the Creative Commons Attribution License (https://creativecommons.org/licenses/by/4.0)
